# Dynamic Changes in Red Cell Distribution Width Can Predict Major Adverse Cardiovascular Events after PCI in Patients with Unstable Angina Pectoris: A Retrospective Cohort Study

**DOI:** 10.1155/2022/2735717

**Published:** 2022-06-08

**Authors:** Qiang Xiao, Dekai Yan, Jian Qin, Wenwen Chen, Ke Jiang, Jian Zhao, Chen Zhang, Yuanmin Li

**Affiliations:** ^1^Department of Cardiology, The Second Affiliated Hospital, Shandong First Medical University & Shandong Academy of Medical Sciences, Taian, China; ^2^Department of Cardiology, The Fourth People's Hospital of Jinan, Jinan, China; ^3^Department of Radiology, the Second Affiliated Hospital, Shandong First Medical University & Shandong Academy of Medical Sciences, Taian, China

## Abstract

**Background:**

The increased red cell distribution width (RDW) is related to a higher risk for cardiovascular disease (CVD). However, it is yet unclear whether the dynamic change of RDW is associated with the major adverse cardiovascular events (MACEs) for individual with CVD.

**Methods and Results:**

A cohort study was conducted among 228 patients who had unstable angina (UA) and underwent PCI. RDW was measured preceding PCI and re-measured on the 16th week after PCI. The change of RDW values was defined as *Δ*RDW. The patients were divided into 3 groups in accordance with *Δ*RDW: improved, stable, and worsened RDW groups. The patients were followed up for 6 years, and MACE episodes were recorded. The survival analysis showed that the incidence of MACEs in stable RDW group was significantly lower than that in improved and worsened RDW groups. By the COX model, the risk of the occurrence of cardiovascular events in improved RDW group was 1.661 times higher than the risk in stable RDW group (HR =1.661, 95% CI: 1.583-2.880, *p* < 0.05) and the same situation was 3.307 times higher in worsened RDW group (HR =3.307, 95% CI: 1.830-5.041, *p* < 0.05).

**Conclusion:**

The measurement of *Δ*RDW has potential to predict the MACEs in UA patients underwent PCI. The dynamic changes in RDW are associated with the outcome of CVD.

## 1. Introduction

In recent years, the incidence and mortality of coronary heart disease (CHD) have been increasing annually [1]. It has become the primary medical cause of mortality in China with a trend of younger affected patients [2]. Unstable angina (UA) is one of acute coronary conditions, usually with worse symptoms, a higher recurrent frequency, and a higher risk of progression to acute myocardium infarction compared to other types of angina. The red blood cell distribution width (RDW) is a parameter reflecting the size heterogeneity of the periphery red blood cells exactly and objectively. For the past few years, there has been a great work of literature reporting a very close relationship between RDW and the occurrence of cardiovascular diseases (CVD) such as cardiac failure and acute coronary syndrome [3, 4]. When RDW keeps a high numerical value, there is commonly a worse coronary artery disease and a big number of diseased vessels. Therefore, the value of RDW is currently used to predict the severity and evaluate prognosis of CVD [5]. The underlying mechanism of RDW and CVD is inflammatory response. Recently, a series of literatures have been introduced and discussed the role of biomarkers such as NLRP3, MTHFR, and palifermin released during inflammatory disease and treatment [6–8]. Meanwhile, there has been no report about the relationship between the distribution width change of red blood cells (*Δ*RDW) following treatment and the afterward risk of CVD. As a novel indicator with predictive value for the diagnosis and treatment of multiple diseases [9], *Δ*RDW may be used to provide the reactivity to CVD treatment. Due to the typicality and universality of unstable angina, the patients with UA were recruited for the study to explore whether *Δ*RDW can be used as an indicator of the CVD risk stratification and long-term prognosis.

## 2. Materials and Methods

### 2.1. Study Patients

**Figure 1:**
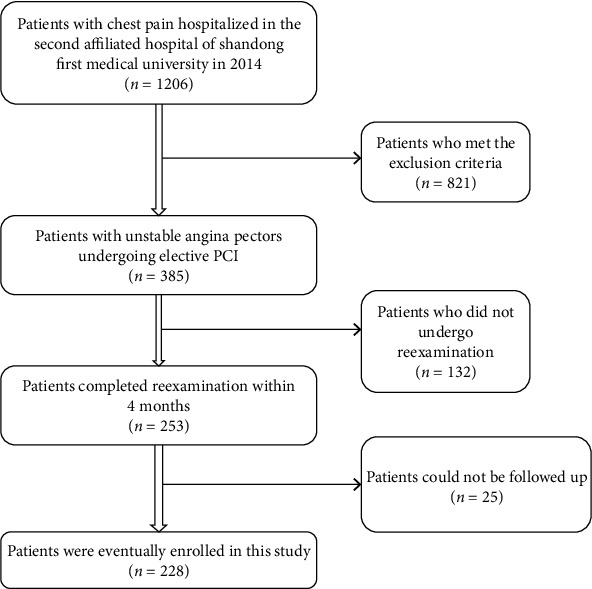
Study flow chart.

A retrospective cohort study was conducted. 228 patients with UA who underwent percutaneous coronary intervention (PCI) in the Second Affiliated Hospital of Shandong First Medical University in 2014 were recruited (Figure 1). The diagnostic criteria of UA conform to the third universal definition of myocardial infarction to define unstable angina of American College of Cardiology (ACC) in 2012 and ESC guidelines on the diagnosis and treatment of Non-ST-Segment Elevation Acute Coronary Syndrome in 2020.

The exclusion criteria include the patients with (1) stable angina pectoris or acute myocardial infarction; (2) history of the disease of gastrointestinal bleeding, anemia, or other bleeding events within one year and during the follow-up period; (3) cardiac insufficiency grading III-IV with NYHA; (4) history of valvular heart disease, cardiomyopathy, pericardial disease, or receiving an operation of pericardial disease; (5) chronic obstructive pulmonary disease (COPD); (6) concurrence of acute and chronic infectious diseases or hematological system diseases; (7) serious hepatic dysfunction or renal insufficiency; (8) concurrence of rheumatism and other autoimmune diseases; (9) hyperthyroidism or hypothyroidism; (10) history of a cancer, receiving chemotherapy, radiotherapy, or organ grafting; (11) a recent blood transfusion or blood donation; (12) no complete data and records.

This study has been approved by the Ethics Committee of the Second Affiliated Hospital of Shandong First Medical University. The investigation conformed to the principles outlined in the Declaration of Helsinki and the informed written consent was given prior to the inclusion of subjects in the study.

### 2.2. Demographic and Clinical Data Collection

In this study, we collected the patients' gender, age, smoking history, alcohol consumption history, hypertension history, and diabetes history. Grace score was used as an index. The diagnostic criteria of hypertension are in accordance of Standard of Guidelines for the Prevention and Treatment of Hypertension in China, by systolic pressure over 140 mmHg and/or diastolic pressure over 90 mmHg in a quiescent condition for two continuous readings or diagnosed hypertension with intake of antihypertensive drugs. The diagnostic criteria of DM are conformed to the Standard of Guidelines for T2DM Prevention and Treatment in China by fasting blood-glucose over 7.1 mmol/L or 2-hour postprandial blood-glucose over 11.1 mmol/L, or diagnosed DM with intake of hypoglycemic agent. The history of alcohol consumption is defined as taking alcohol over 100 g each day lasting for a year or longer; the smoking history is defined as smoking at least one cigar over a year or longer.

### 2.3. Testing Method and Index

#### 2.3.1. Blood Testing Method

(1) Venous blood was drawn following fasting for 8 hours after admission; (2) fasting venous blood was drawn after fasting for 8 hours when patients visited the hospital for reexamination within 4 months after discharge. All blood samples were transferred to the hospital laboratory and all tests were operated by the professional staffs.

#### 2.3.2. Testing Index

The testing indexes, such as red blood cell count (RBC), red blood cell distribution width (RDW), white blood cell count (WBC), blood platelet count (PLT), hemoglobin (Hb), mean corpuscular volume (MCV), fasting blood-glucose (FBG), total cholesterol (TC), triglyceride (TG), low-density lipoprotein (LDL-C), high-density lipoprotein (HDL-C), creatinine (SCr), blood urea nitrogen (BUN), *β*2-microglobulin (*β*2-MG), and uric acid (UA), were obtained.

In this study, a patient's RDW numerical value upon admission for PCI was recorded as RDW1, while the value of the same patient within 4 months after discharge was recorded as RDW2. The difference of two RDW numerical values was calculated and recorded as *Δ*RDW, then the trisection method was used to divide the patients into improved RDW group, stable RDW group, and worsened RDW group.

### 2.4. PCI Procedure and Interpretation of Results

All subjects underwent PCI and radial artery catheterization was adopted by Seldinger method during hospitalization. The PCI procedure and results evaluation were performed by 2-3 cardiovascular specialists, and clinical risk stratification was conducted using GRACE risk scoring systems.

### 2.5. Clinical Follow-up

All 228 patients included in this study were followed up regularly starting on February 5, 2014, and ending on December 18, 2020. The patients were followed up through telephone calls, outpatient visits, and review of inpatient medical records. Collected information mainly included the major adverse cardiovascular events (MACE) during the follow-up period, the time of event occurrence, and the patient's medical treatment. MACEs are defined as revascularization, acute myocardial infarction, malignant arrhythmias, ischemic stroke, and death. The MACEs during follow-up were recorded in all three groups.

### 2.6. Statistical Method

Statistical analysis was conducted on SPSS22.0 software, and *p* < 0.05 was considered statistically significant. The Kolmogorov-Smirnov test was used in normality analyses of the study variables. The normal distribution of measurement data was expressed as mean ± standard deviation ($\overset{-}{x}\pm s$). One-way ANOVA was used for comparison between groups. The non-normal distribution of measurement data was expressed as median and interquartile range; and the comparison between groups was performed by rank sum test. Enumeration data were expressed by frequency or percentage, and comparison between groups was performed by *χ*^2^ test. The Kaplan-Meier curve analysis was performed for the three groups of MACE, while the *Δ*RDW predictive effect on MACE in UA patients after PCI treatment was analyzed by a Cox proportional hazards model.

## 3. Results

### 3.1. Comparison of Demographic and Clinical Data among 3 Groups

According to the *Δ*RDW values of 228 patients aligned from low to high, the trisection method was used to divide the patients into improved RDW group (-2.4~-0.5) of 80 patients, stable RDW group (-0.49~0.2) of 80, and worsened RDW group (0.21~3.6) of 68. There were statistically significant differences in the histories of alcohol consumption and hypertension among the three groups (*p* < 0.05). There were no significant differences in GRACE score, gender, age, systolic blood pressure, diastolic blood pressure, smoking history, diabetes history, ACEI drugs, ARB drugs, *β* drugs, CCB drugs, statin use, and the duration of dual antiplatelet therapy (DAPT) among the three groups (*p* > 0.05). The data are summarized in Table 1(a). The detail in incidence of MACE in the three groups is shown in Table 2. Since the stable RDW group includes patients with low RDW1-low RDW2 and patients with high RDW1-high RDW2, there were no significant differences in the risk of cardiovascular events between the two subgroups (*χ*^2^ = 0.033, *p* = 0.085) (Table 3).

### 3.2. Comparison of Lab Indexes among 3 Groups

There were statistically significant differences in Hb, PLT, and BUN among the three groups (*p* < 0.05). The comparison of RBC, WBC, MCV, GLU, TC, TG, LDL-C, HDL-C, SCr, and *β*_2_-MG revealed no statistical significance. There was no significant difference in baseline values of RDW between the three groups, whether values of RDW1 or RDW2 (*p* > 0.05) (Table 1(b)).

### 3.3. Kaplan-Meier Curve Analysis of MACE and *Δ*RDW

**Figure 2:**
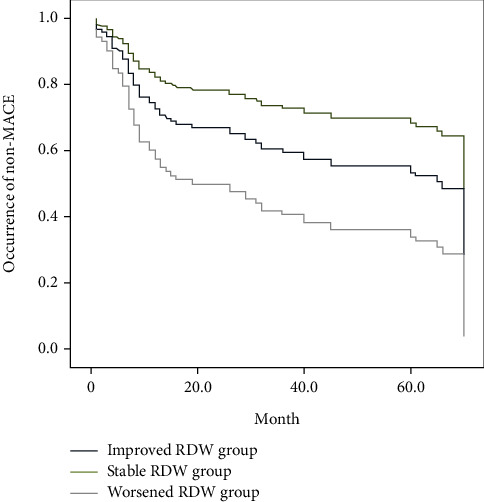
The Kaplan-Meier curve analysis of 3 groups.

To assess the completeness of the follow-up, He Person-Time Follow-up Rate (PTFR) was calculated using a simplified person-time method (SPT) [10]. Consequently, the PTFR was 74.78% in our follow-up. Assigning numbers to *Δ*RDW as an independent variable, improved RDW group = 1, stable RDW group = 2, worsened RDW group = 3. Taking MACE and the follow-up duration as dependent variables, MACE = 1, No MACE = 0, the follow-up duration = t using unit of month. A Kaplan-Meier survivorship curve was employed to analyze the relationship between *Δ*RDW and MACE. Log Rank (Mantel-Cox), Breslow (Generalized Wilcoxon), and Tarone-Ware tests all demonstrated that the survivorship difference among 3 groups is statistically significant (*χ*^2^ = 21.149, *p* = 0.01), as shown in Table 4. The Kaplan-Meier survivorship curve showed that the cumulative incidence of MACE in 6 years in stable RDW group was clearly lower than the occurrence rates in improved RDW group and worsened RDW group (Figure 2).

### 3.4. Regression Analysis of Cox Proportional Hazards Model

The dependent variable was assigned with a number (occurrence of MACE = 1; no-occurrence of MACE = 0). The covariables were also assigned including the different groups of *Δ*RDW (improved RDW = 1, stable RDW =2, and worsened RDW =3), hypertension history (Yes = 1, No = 0), diabetes mellitus (Yes = 1, No = 0), alcohol consumption history (Yes = 1, No = 0), smoking history (Yes = 1, No = 0), gender (Make = 1, Female = 0), age, TC, TG, Hb, PLT, UA, BUN, LDL-C, HDL-C, RDW1 (basal RDW value), and GRACE score. The risk analysis suggested that basal RDW value (HR: 1.391, 95% CI: 1.124 to 1.721, *p* = 0.002) and GRACE score (HR: 1.050, 95% CI: 1.033 to 1.066, *p* < 0.001) were the risk factors that came into the equation. Using stable RDW group as a reference, the risk of the occurrence of cardiovascular events in improved RDW group was 1.661 times higher than the risk in stable RDW group (HR =1.661, 95% CI: 1.583-2.880, *p* < 0.05) and the same situation was 3.307 times higher in worsened RDW group (HR =3.307, 95% CI: 1.830-5.041, *p* < 0.05). In a conclusion, stable *Δ*RDW is a protective factor in MACE, and potentially acts as a long-term independent predictive factor for the patients with unstable angina pectoris after PCI treatment.

## 4. Discussion

Some studies [11, 12] have focused on the relationship between baseline level of RDW and the MACE in patients undergoing PCI, rather than the change value of RDW before and after intervention. This is precisely the feature and highlight of our research.

Different from previous studies having focused on the functional changes of platelets, recent studies have shown that abnormal structure and function of red blood cells are involved in the occurrence and development of cardiovascular diseases, and become a novel target for treatment and prognosis of coronary heart disease (CHD) [13]. In fact, erythrocyte reactivity, or the altered function of RBCs, is an area of concern after treatment, especially PCI [14]. RDW is one of the indices of routine blood test, and reflects the dispersion degree of peripheral red cell size [15]. Changes in RDW are thought to be an aspect of RBC reactivity [16]. Many studies have found that increased RDW is a poor prognostic indicator of a variety of cardiovascular diseases, and used in risk stratification [17–20].

Various factors are involved in the occurrence and development of CHD, including hypertension, hyperlipidemia, hyperglycemia, smoking, obesity, inflammation, uric acid, and other risk factors. Some scholars have shown that the elevated RDW value may be related to chronic inflammation, oxidative stress response, and neuroendocrine dysfunction [21, 22]. Coronary atherosclerosis is the basis of the occurrence and development of CHD. Inflammation can not only increase RDW but also is an important mechanism of atherosclerosis. Inflammation and oxidative stress lead to arterial plaque instability, which play an important role in the occurrence and development of CHD.

Relevant studies have shown that erythrocyte aggregation and blood viscosity in patients with CHD are significantly higher than those in normal population [23]. When the total cholesterol in adtevak keeps accumulating, it can enter the lipid layer of erythrocyte membrane, which increases the density of the membrane, restricts the movement of membrane protein molecules, reduces the membrane fluidity, and thus leads to the reduced deformability of the erythrocyte [15]. The reduction of erythrocyte deformability and enhancement of erythrocyte aggregation can further increase the whole blood viscosity and lead to tissue ischemia and hypoxia. The ischemia and hypoxia developed in the myocardial tissue when coronary artery stenosis is present activates RASS system and increases the angiotensin II level, motivates the secretion of erythropoietin, and consequently irritates erythropoiesis [24]. The newborn red blood cells are different from the mature erythrocytic shape and size to a certain degree, and further raise the RDW level. At the same time, it can increase the erythrocyte aggregation and reduce the deformability, further aggravating the tissue ischemia and hypoxia, so that it forms a vicious cycle [25].

The purpose of this study is to discuss the relevance of the change of RDW (*Δ*RDW) in patients with unstable angina (UA) following PCI treatment in the MACE. The results showed that the occurrence rate of MACE in patients with stable RDW was clearly smaller than the other two groups. When the numeral value of RDW rises, it means the volume otherness of red blood cells increases; when the numeral value of RDW is smaller, on the other side, the volume otherness of red blood cells is lower. Therefore, the smaller as the RDW change is, the more stable the RBC volume is, indicating that the RBC response is neutral to treatment.

Stable RDW is a protective factor of MACE to patients with UA receiving PCI, through the mechanisms discussed above. In stable RDW group, the red blood cell volume is relatively stable, that is, the aggregation and deformability of erythrocytes are relatively steady. Also, all patients in this study were treated with statins for lipid-lowering therapy. The effects of statins on stabilizing plaque, inhibiting inflammation, and delaying atherosclerosis may contribute to the protection as well. *Δ*RDW represents the changing scope of the volume of red blood cell in a fixed period time, which reflects a more real condition of the long-term prognosis of CVD than the simple comparison between the basic numeral values of RDW. Nevertheless, its specific mechanism and accuracy as a predictive and prognostic marker still need large-scale and long-term research.

In conclusion, changes in RDW, or RBC reactivity, are significantly associated with MACE events in UA patients after PCI treatment. Stable RDW is a protective factor of MACE. *Δ*RDW may be an independent predictive factor of MACE in unstable angina pectoris following treatment.

There are some limitations in this study. Among them, considering the indications of stenting for stable angina and the inconvenience of rescuing acute myocardial infarction, only patients with unstable angina were recruited into the study, so there was a certain selection bias. Furthermore, the patient size was overall small, the follow-up duration was relatively short, and the inflammatory indexes on admission were not included in the data collection, which will be further improved in the future work. In addition, it is also interesting to discuss whether *Δ*RDW can be used as an early biomarker of disease change, similar to the role of MTHFR in assessing periodontitis control [7].

## Figures and Tables

**Table:** (a) Comparison of general data among 3 groups

Variables	Improved RDW group (*n* = 80)	Stable RDW group (*n* = 80)	Worsened RDW group (*n* = 68)	*F*/*χ*^2^	*p* value
Gender/male	46 (57.50)	54 (67.50)	40 (58.80)	1.960	0.375
Age (years)	61.00 (9.00)	60.50 (16.00)	63.00 (9.00)	3.33	0.180
Smoking	32 (40.00)	40 (50.00)	26 (38.20)	2.523	0.283
Drinking	28 (35.00)	36 (45.00)	18 (26.5)	5.530	0.043
Hypertension	46 (57.50)	50 (62.50)	52 (76.50)	6.123	0.047
Diabetes	18 (22.50)	24 (30.00)	14 (20.60)	2.040	0.361
Duration of DAPT	70 (87.50)	74 (92.50)	64 (94.10)	2.261	0.323
ACEI	40 (50.00)	32 (40.00)	34 (50.00)	2.087	0.352
ARB	8 (10.30)	16 (20.00)	14 (20.60)	3.671	0.160
*Β*-blocker	54 (67.50)	50 (62.50)	46 (67.60)	0.593	0.743
CCB	26 (32.50)	24 (30.00)	20 (29.40)	0.193	0.908
Statin	74 (92.50)	75 (93.75)	64 (94.12)	0.993	0.841
LVEF (%)	55.71 ± 7.62	54.93 ± 5.33 (19.50)	56.36 ± 8.69	1.137	0.566
SBP (mmHg)	137.50 ± 12.26	138.21 ± 12.19 (10.00)	135.93 ± 11.82	1.316	0.518
DBP (mmHg)	79.89 ± 13.53	81.00 ± 15.11 (19.50)	78.93 ± 12.89	4.498	0.105
Grace scores	97.40 ± 14.66	98.51 ± 16.76	97.91 ± 15.97	4.164	0.119

ACEI: angiotensin-converting enzyme inhibitors; ARB: angiotensin receptor blockers; CCB: calcium channel blocker; DAPT: dual antiplatelet drug therapy; DBP: diastolic blood pressure; LVEF: left ventricular ejection fraction; RDW: red cell distribution width; SBP: systolic blood pressure.

**Table:** (b) Comparison of lab indexes among 3 groups

Variables	Improved RDW group (*n* = 80)	Stable RDW group (*n* = 80)	Worsened RDW group (*n* = 68)	*F*/*χ*^2^	*p* value
Hb (g/L)	133.5 (18.5)	150.0 (10.0)	118.5 (8.0)	173.509	0.021
RBC (×10^12^/L)	4.36 ± 0.41	4.35 ± 0.44	4.28 ± 0.50	0.646	0.525
WBC (×10^9^/L)	5.95 (2.35)	6.38 (3.01)	5.73 (3.30)	1.354	0.508
PLT (×10^9^/L)	220.50 (71.25)	207.00 (76.25)	198.50 (89.00)	9.203	0.010
MCV (fl)	92.00(6.00)	91.25 (7.00)	91.50 (7.90)	4.161	0.125
TC (mmol/L)	4.69 ± 1.15	4.44 ± 1.02	4.54 ± 1.23	0.993	0.372
TG (mmol/L)	1.42 (1.05)	1.20 (0.81)	1.16 (0.84)	4.374	0.112
HDL-C (mmol/L)	1.12 (0.38)	1.16 (0.23)	1.19 (0.43)	2.856	0.240
LDL-C (mmol/L)	2.61 (1.30)	2.47 (1.08)	2.46(1.42)	1.201	0.549
ApoA (g/L)	1.65 ± 0.26	1.48 ± 0.13	1.96 ± 0.39	4.341	0.079
ApoB (g/L)	0.91 (0.44)	0.84 (0.37)	0.86 (0.47)	1.617	0.446
FPG (mmol/L)	5.48 (1.83)	5.23 (0.98)	5.27 (1.49)	2.608	0.271
BUN (mmol/L)	5.13 (1.82)	5.55 (1.84)	4.85 (2.40)	8.241	0.016
SCr (umol/L)	67.60 ± 11.33	71.40 ± 15.66	69.79 ± 14.84	1.479	0.230
*β*2-MG (mg/L)	2.29 (0.71)	2.21 (0.69)	2.30 (0.51)	1.324	0.511
Cys-c (mg/L)	0.86 ± 0.17	0.84 ± 0.21	0.85 ± 0.16	0.193	0.825
RDW1	13.70 ± 0.91	12.67 ± 0.78	12.62 ± 1.02	4.165	0.611
RDW2	12.57 ± 0.60	12.61 ± 0.78	13.69 ± 1.21	3.575	0.403

ApoA: apolipoprotein A; ApoB: apolipoprotein B; BUN: blood urea nitrogen; *β*2-MG: *β*2-microglobulin; Cys-c: cystatin C; FPG: fasting plasma glucose; Hb: hemoglobin; HDL-C: high-density lipoprotein cholesterol; LDL-C: low-density lipoprotein cholesterol; MCV: mean corpuscular volume; PLT: platelet; RBC: red blood cell; RDW: red blood cell distribution width; TC: total cholesterol; TG: triglycerides; WBC: white blood cell.

**Table 2:** The distribution frequency of MACEs in the 3 groups.

Group	Revascularization	Acute myocardial infarction	Malignant arrhythmias	Ischemic stroke	Death
Improved RDW group (*n* = 80)	24	3	2	6	1
Stable RDW group (*n* = 80)	26	1	1	4	0
Low RDW1-low RDW2 (*n* = 41)	14	0	1	1	0
High RDW1-high RDW2 (*n* = 39)	12	1	0	3	0
Worsen RDW group (*n* = 68)	30	3	2	8	1

**Table 3:** The comparison of MACEs in the 2 subgroups of stable RDW group.

	Non-MACE	MACE	*χ* ^2^	p
Low RDW1-low RDW2 (*n* = 41)	16	25	0.033	0.855
High RDW1-highRDW2 (*n* = 39)	16	23		

**Table 4:** Comparison of survivorship analysis in general.

	*χ* ^2^	df	*p* value
Log Rank (Mantel-Cox)	21.149	2	0.001
Breslow (Generalized Wilcoxon)	23.548	2	0.001
Tarone-Ware	22.897	2	0.001

## Data Availability

The data used to support the findings of this study are restricted by the Ethics Committee of the Second Affiliated Hospital of Shandong First Medical University in order to protect PATIENT PRIVACY. Data are available from Yuanmin Li (liym575@126.com) for researchers who meet the criteria for access to confidential data.
